# No further gain can be achieved by calculating Disease Activity Score in 28 joints with high-sensitivity assay of C-reactive protein because of high intraindividual variability of C-reactive protein: A cross-sectional study and theoretical consideration

**DOI:** 10.1097/MD.0000000000005781

**Published:** 2017-01-10

**Authors:** Inger M.J. Hansen, Amir Emamifar, Rikke A. Andreasen, Steen Antonsen

**Affiliations:** a Department of Rheumatology, Svendborg Hospital, Odense University Hospital, Svendborg; b University of Southern Denmark, Odense; c Danbio, Copenhagen; d Department of Clinical Biochemistry, Svendborg Hospital, Odense University Hospital, Svendborg, Denmark.

**Keywords:** C-reactive protein, DAS28, intraindividual biological variation, reporting limit, rheumatoid arthritis

## Abstract

Disease Activity Score in 28 joints (DAS28) is commonly used to evaluate disease activity of rheumatoid arthritis (RA) and is a guide to treatment decision.

The aim of this study was to evaluate the impact of lower reporting limit for C-reactive protein (CRP), with respect to intraindividual biological variability, on the calculation of DAS28 and subsequent patient classification.

This study consists of 2 sections: a theoretical consideration discussing the performance of CRP in calculating DAS28 taking intraindividual biological variation and lower reporting limit for CRP into account and a cross-sectional study of RA patients applying our theoretical results. Therefore, we calculated DAS28 twice, with the actual CRP values and CRP = 9 mg/L, the latter to elucidate the positive effects of reducing the lower reporting limit of CRP from <10 to <3 mg/L.

Lower-reporting limit of <10 mg/L leads to overestimate DAS28. However, reducing lower reporting limit for CRP to <3 mg/L results in optimizing DAS28 calculation. Further lowering of reporting limit for CRP to <3 mg/L does not increase the precision of DAS28 owing to the relatively large intraindividual biological variation.

Five hundred twelve patients were included. There was a significant difference between recalculated and patients DAS28 (*P* < 0.001). One hundred nine patients had DAS28 deviation (compatible to remission to low: 66, low to moderate: 39. and moderate to high: 4).

Owing to significant impact of intraindividual biologic variation on DAS28 and patient classification, special attention should be paid to calculate DAS28 when CRP values are within normal range. Furthermore, we conclude that results of different studies evaluating DAS28 and treatment response are not comparable if the reporting limits of CRP are unknown.

## 1 Introduction

Rheumatoid arthritis (RA) is a chronic disease with a prevalence of 0.5% to 1% of the general population. Pain, swelling, redness, fatigue, decreased functional capacity and prolonged stiffness following rest are the most frequent symptoms. It has been generally accepted that RA should be treated instantly to improve clinical outcome and prevent further joint destruction. In order to achieve this goal, various composite indices have been proposed to monitor RA disease activity and effects of treatment ^[1–3]^.

Disease activity score (DAS) is a scoring system that is commonly used in daily clinical practice to evaluate disease activity of RA.^[4]^ It was firstly derived from a set of 4 variables including: number of painful joints calculated with the Ritchie Articular Index, 44-joint-count for swelling, erythrocyte sedimentation rate (ESR), and general health or patient's global assessment (PGA) of disease activity on a 100-mm visual analog scale (VAS). Subsequently, DAS28 was developed and validated.^[3]^ The DAS28 score is calculated from 4 components including: tender joints (TJ)(range 0–28), swollen joints (SJ) (range 0–28), PGA (range 0–100), and laboratory values of ESR or C-reactive protein (CRP).^[5]^ DAS28-CRP and DAS28-ESR can be calculated using the following, closely related formulas: DAS28-CRP = 0.56∗√(TJ) + 0.28∗√(SJ) + 0.36∗ln(CRP+1) + 0.014∗PGA + 0.96 and DAS28-ESR = 0.56∗√(TJ) + 0.28∗√(SJ) + 0.70∗ln(ESR) + 0.014∗PGA. In the authors’ country, Denmark, DAS28-CRP is generally used to measure disease activity. DAS28-CRP ranges from 0.96 to maximum of 9.4 (the latter if CRP is 100 mg/L). A DAS28 value of >5.1 indicates high disease activity, 3.2 <DAS28 ≤5.1 and DAS28 ≤3.2 are defined as moderate and low disease activity. Patients may be considered to be in remission phase if DAS28 is <2.6.^[6]^

CRP is an acute-phase reactant that increases within 4 to 6 hours of inflammation or acute tissue injury.^[7]^ It can be tested by different assays with different measuring ranges. Previously, lower measuring limits of 3 to 10 mg/L were common, whereas during recent years, analytical methods optimized to measure results as low as 0.1 mg/L have become widely used, often called high-sensitivity CRP (hs-CRP) methods. The threshold for reporting of CRP has in most hospitals been declining over time at a varying speed and with variations from laboratory to laboratory. The potential clinical benefits of measuring CRP in the normal range using hs-CRP methods have been evaluated broadly in cardiovascular disease,^[8,9]^ whereas the role of hs-CRP in rheumatoid diseases has been investigated to much lesser extent.

The intraindividual biological variation (CV-within) of CRP in healthy subjects has been estimated to 42% to 76%, whereas analytical variation is <10%.^[10,11]^ Furthermore, near lower reporting limit, the intraindividual biological variation of CRP in RA patients is unknown; however, it is probably higher than in healthy subjects with identical mean CRP level. Even if it should be lower than for healthy subjects, it is still a factor of variation with major influence on the DAS28. We showed previously that biological variation of CRP might cause significant fluctuation in the DAS28 score with consequence on patient classification and subsequent treatment plan.^[12]^

With respect to DAS28 and RA, reporting limit for CRP becomes more important, as CRP has a direct effect on calculating DAS28, patient classification, and treatment decisions. The objective of present study is to investigate the impact of lower reporting limit for CRP, taking intraindividual biological variation into account, to calculate DAS28 and subsequent patient classification both theoretically and clinically. In our hospital, CRP results <10 mg/L were reported as <10 mg/L until 25th February 2013. In the period from February 25, 2013 to March 17, 2015, results were reported down to 3 mg/L and lower results as <3 mg/L. Since March 17, 2015 results of CRP are reported down to 0.6 mg/L.

## 2 Materials and method

The present study consists of 2 sections: the first section is a theoretical consideration discussing the performance of CRP in calculating DAS28 with regard to intraindividual biological variation and the reporting limit for CRP (Part A and B); the second section is a cross-sectional study to evaluate the clinical significance of the lower reporting limit for CRP in all our RA patients seen until April 2015.

### 2.1 Theoretical consideration

#### 2.1.1 Part A

**Figure 1:**
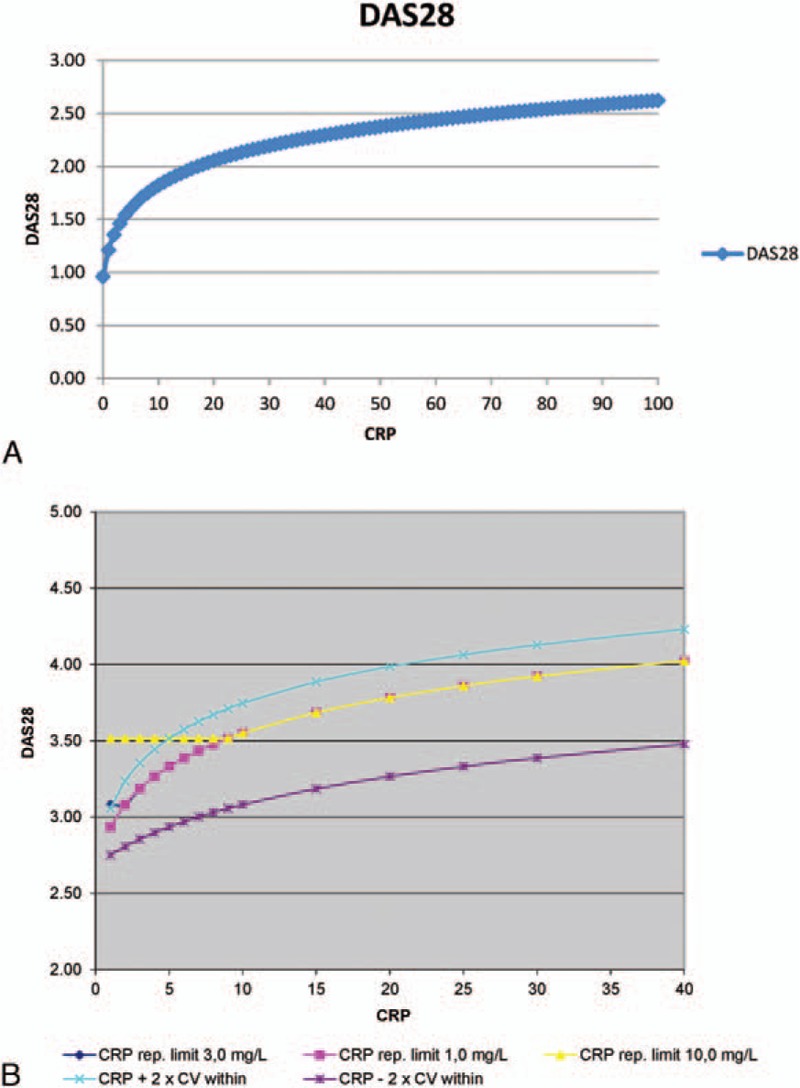
(A) The relation between DAS28 and CRP, when CRP ranging from 0 to 100 and other parameters were held constant (Tender joint = 0, Swollen joint = 0, and patient global assessment = 0). (B) Dependency of DAS28 to CRP (theoretical consideration). Tender joint: 3, Swollen joint: 1, and patient global assessment: 34. CV-within: intraindividual biological variation. DAS28 = Disease Activity Score in 28 joints, CRP = C-reactive protein.

The aim of this study was to evaluate the isolated performance of CRP values to calculate DAS28, in particular when CRP is <10 mg/L. We calculated DAS28 considering single parameter (CRP) with holding other parameters zero by using the following formula: DAS28 = 0.56∗√(TJ) + 0.28∗√(SJ) + 0.36∗ln(actual CRP value+1) + 0.014∗PGA + 0.96. Therefore, different values of CRP ranging from 1 to 100 mg/L plus TJ = 0, SJ = 0, and PGA = 0 were entered into the formula to perform the calculation (Fig. 1A).

#### 2.1.2 Part B

With a lower reporting limit of CRP <10 mg/L, the real CRP values could be all figures between 9 and 0 and with a lower reporting limit of CRP <3 mg/L, between 2 and 0 as well. As a result, we introduced the following example with the lower limit of <10 mg/L and 3 mg/L taking into account the intraindividual biological variation.

Different values of DAS28 based on various values of CRP (0–40 mg/L) and constant values of TJ (TJ = 3), SJ (SJ = 1), and PGA (PGA = 34) were calculated using the above-mentioned DAS28 formula, as well as upper and lower limits of DAS28. Thus, DAS28 were calculated 5 times as described below:

1. With CRP values ranging from 1 to 40 mg/L without limit.
2. With CRP values with lower reporting limit of 3 mg/L ranging from 3 to 40 mg/L.
3. With CRP values with lower reporting limit of 10 mg/L ranging from 10 to 40 mg/L.
4. With upper limit of CRP based on biological variation. The upper limit values of CRP were calculated as CRP plus CRP multiplied by the lowest percentage (42%) of the intraindividual biological variation previously described, multiplied with 1.96 (twice the standards deviation [SDs]) (CRP + [CRP × 0.8]).
5. With lower limit of CRP values based on biological variation. The lower limit values of CRP were calculated as CRP minus CRP multiplied by the lowest percentage (42%) of the intraindividual biological variation previously described, multiplied with 1.96 (twice the SD) (CRP − [CRP × 0.8]).

A line chart was drawn to show the results of different DAS28 values (Fig. 1B).

### 2.2 Cross-sectional study

The Danish Danbio registry provides national data on the disease course of patients with inflammatory rheumatic disease including RA via a personal identification number. It was established in 2000 and is approved by the Danish Data Registry (j. nr. 2007–58–0014 and j.nr. 2007–58–0006) and National Board of Health (j. nr. 7–201–03–12/1). Since 2007, all newly diagnosed RA patients in Denmark should be registered in Danbio. Variables in Danbio are categorized into baseline and longitudinal.^[13]^ At our rheumatology outpatient clinic, these variables for our entire RA patients are entered into Danbio at every consultation.

### 2.3 Setting, design, and ethics committee approval

This was a-cross sectional, registry-based, single center study. All parts of study were performed at the rheumatology outpatient clinic.

Ethical approval was obtained from Danish Data Protection Agency (file no.15/25403).

### 2.4 Participants

All patients diagnosed with RA are registered in the local part of Danbio by rheumatologists during outpatient visits. In April 2015, data for our entire RA patients (n = 876) were extracted from Danbio. Since 2010, diagnosis of RA was made according to the new 2010 American College of Rheumatology (ACR)/European League against Rheumatism (EULAR) criteria for RA.

Inclusion criteria were as follows: patients who were registered in the rheumatology outpatient clinic, age ≥18 years, CRP results were reported after 25th Feb 2013 since lower reporting limit was set to 3 mg/L and then 0.6 mg/L. Patients who passed away or were referred to the other departments were also included. Patients with incomplete Danbio registration were excluded from the study.

After obtaining all data from included patients, DAS28 was calculated for each individual patient twice, first with patients actual CRP and hereafter with a constant value of CRP = 9 mg/L to illustrate the consequences of the lower reporting limit of CRP for the DAS28 calculation. Subsequently, we assessed whether new DAS28 values caused any changes in the patients classification.

### 2.5 Data collection and variables

Patients’ demographic data including age, sex, and disease characteristics (TJ, SJ, PGA, DAS28, CRP, IgM rheumatoid factor [IgM RF] [normal range: <15 IU/mL], and anti-cyclic citrullinated peptide [anti-CCP] [normal range: <20 EU/mL]) were extracted from Danbio. DAS28 was calculated using the following formula: DAS28 = 0.56∗√(TJ) + 0.28∗√(SJ) + 0.36∗ln(CRP+1) + 0.014∗PGA + 0.96 with actual CRP initially and CRP = 9 mg/L thereafter. To measure PGA, 100-mm visual analogue scale technique was used.

### 2.6 Statistical analysis

All statistical analyses were performed by Microsoft Excel 2010. Continuous data are presented as mean ± SD, categorical data as frequencies and respective percentages. We used following ranges to indicate the patients’ disease activity: high disease activity DAS28 >5.1, moderate disease activity 3.2 < DAS28 ≤ 5.1, low disease activity DAS28 ≤3.2, and remission phase compatible with DAS28 <2.6. *P* values were calculated using the Student *t* test and considered as significant if *P* < 0.05. Number of patients with changes in disease activity classification has been shown with bar chart. In case of missing data, we used pairwise deletion to keep as many cases as possible for each analysis.

## 3 Results

### 3.1 Theoretical consideration

#### 3.1.1 Part A

Calculating DAS28 holding TJ, SJ, and PGA zero and increasing CRP from 0 to 100 mg/L shows that there is a steep increase in DAS28 specially when CRP <10 mg/L and to a lesser extent when CRP = 10 to 20 mg/L. The area of CRP between 0 and 10 mg/L is of particular interest in this article because of the lower reporting limit for CRP within this range. The steepness of the curve is because of the logarithmic function of CRP when calculating DAS28 (ln [CRP + 1]) (Fig. 1A).

#### 3.1.2 Part B

Figure 1B illustrates the DAS28 with varying CRP levels, whereas other contributors to DAS28 (TJ, SJ, and PGA) are held constant at arbitrarily chosen values. Furthermore, lines of CRP ± 1.96 × CV-within are shown. A single CRP result of an individual person will with a probability of 95% be within these limits. In case of lower reporting limit of 10 mg/L and interpreting all results of <10 mg/L as 9 mg/L results is shown as the curve marked by triangles, which cross CRP + 2 × CV-within around 6 mg/L, meaning that DAS28 will be overestimated, more than could explained by intraindividual biological variation, when CRP is really <5 mg/L.

If lower reporting limit was 3 mg/L and all results of <3 mg/L were interpreted as 2 mg/L, results are given as the curve marked by diamonds, which slightly increases CRP + 2 × CV-within at 1 mg/L, meaning that DAS28 will be slightly overestimated, more than explained by intraindividual biological variation when CRP really is ≤1 mg/L.

Lowering of reporting limit for CRP to <3 mg/L cannot be expected to contribute to the precision of DAS28 owing to the relatively large intraindividual biological variation making a huge impact on DAS28.

### 3.2 Cross-sectional study

A total of 876 patients with diagnosis of RA were registered in the local part of Danbio. Three hundred sixty-four patients were excluded from the study. Reasons of exclusion were: incomplete Danbio registration (n = 108) or lack of CRP results and CRP results were reported before February 25, 2013. Of 512 included patients, 332 (65%) were female and the mean of age was 65.5 ± 14.0 (mean ± SD). Demographic and disease characteristics are summarized in Table 1.

**Table 1:**
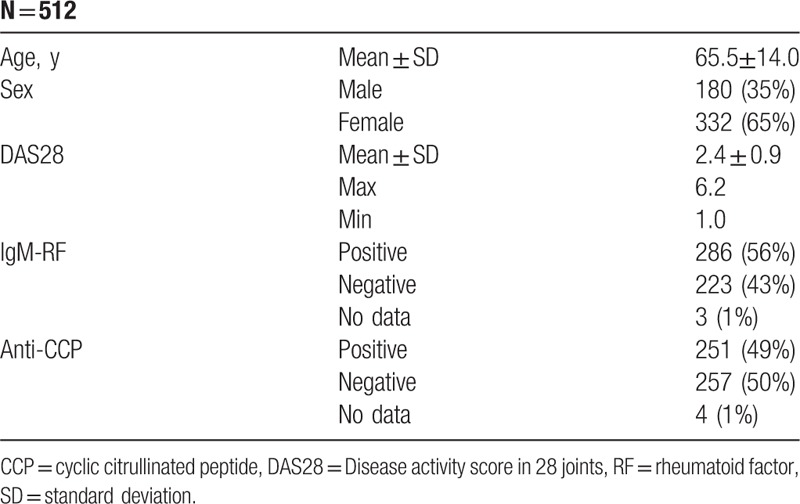
Characteristic of Patients.

The mean value of recalculated DAS28 was 2.8 ± 0.9 (mean ± SD). There was a statistically significant difference between patients’ DAS28 (with actual CRP) and recalculated DAS28 with CRP = 9 mg/L (*P* < 0.001).

**Figure 2:**
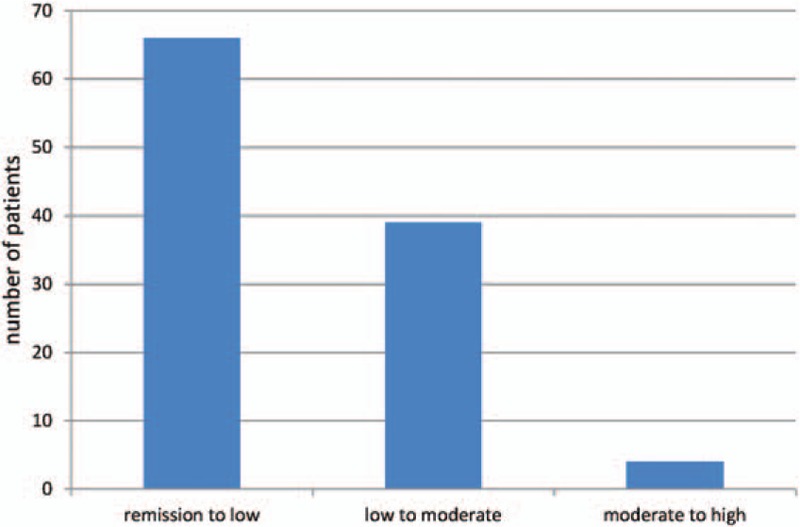
Changes in patients’ disease activity when DAS28 calculated with CRP = 9 mg/L. DAS28 = Disease Activity Score in 28 joints, CRP = C-reactive protein.

Recalculating DAS28 with CRP = 9 mg/L resulted in deviation of patient classification from compatible with remission to low (66 patients), low to moderate (39 patients), and moderate to high (4 patients) (a total of 109 patients) (Fig. 2), illustrating that a reporting limit of <10 mg/L combined with interpretation of results reported as <10 mg/L as 9 mg/L would have resulted in a misclassification of 14% of the patients in an upward direction.

## 4 Discussion

It has been generally accepted that RA should be controlled instantly. Regular clinical evaluation of RA with the aim of different scoring systems including DAS28 helps clinicians to reach the optimal control. DAS28 score is a guide to start, change, or to stop treatment with disease-modifying antirheumatic drugs (DMARDs). Therefore, it should be calculated precisely, whereas miscalculation of DAS28 score results in incorrect patient classification and treatment plan.^[12]^ The key result of this study can be summarized as follows: lower reporting limit of <10 mg/L in combination with interpretation of <10 mg/L as 9 mg/L, leads to inaccurate patient classification in a great number of patients (109/512 in this study) with possibly consequences on treatment decision and this is particularly relevant when initiating of biologics has been considered because of considerable medical expenses; reducing lower reporting limit for CRP to <3 mg/L will optimize the correct patient classification; and there is no further gain from calculating DAS28 with high sensitivity assay of CRP because of high intraindividual variability of CRP. Considering real CRP values between 0 and 9, there is a large impact on final DAS28 score if CRP values are reported as <10 mg/L with a direct effect on patient classification as well as treatment decision. However, reducing the reporting limit for CRP values to <3 mg/L will minimize the impact on DAS28 score and patient classification. This impact is greatest, when CRP values are <10 mg/L because of the logarithmic function of CRP in the formula (0.36∗ln [CRP + 1]). These results were also applicable in the RA population included in the study, as a total of 109 (21.3%) patients had a disease activity deviation from compatible to remission to low, low to moderate, or moderate to high, when DAS 28 was calculated using CRP = 9 mg/L, comparing with actual values of CRP, if the reporting limit has not been reduced to <3 mg/L.

During the last decades, high sensitive assays have been developed to detect lower CRP concentration (down to <0.1 mg/L) than by earlier low-sensitivity laboratory techniques.^[14]^ These assays are commonly used in cardiovascular disease risk assessment and to a lesser extent in other clinical areas especially malignant diseases.^[15,16]^ Based on our theoretical consideration, a lower reporting limit for CRP <3 mg/L will result in lower DAS28 scores; however, these will still be challenged by high intraindividual variability of CRP and cannot be expected to increase the clinical usefulness of DAS28. Thus, for the individual patient, neither risk assessment in various diseases nor DAS28 calculation are optimized using hs-CRP measurements because of the intraindividual biologic variation of CRP. Consequently, the authors do not think that it is necessary to implement high-sensitivity CRP assays for calculating DAS28. To reduce the effect of intraindividual biological variation of CRP, DAS28 might be calculated using the average of multiple CRP measurements in the individual patient taken during a period of stable disease activity. Theoretically, an average of 4 and 9 consecutive CRP measures reduces the intraindividual biological variation by half and one-third; however, it is unrealistic in daily clinical practice.

DAS28, as mentioned above, can be measured by either applying CRP or ESR values. The formulas are used to calculate DAS28-ESR and DAS-CRP are very close to each other. The logarithmic function of ESR and CRP in the formulas causes great fluctuations, especially in the lower values. Therefore, with respect to the intraindividual biological variability (around 30%) and lower reporting limit of ESR (1 mm),^[17]^ it is theoretically feasible to consider similar misinterpretation in the calculation of DAS28-ESR and further misclassification of the patients; however, it is essential to mention that such an assumption is not practically relevant because the reporting limit of ESR has not changed since the establishment of ESR analysis in the 19^th^ century.^[18]^ However, results of the present study potentially affect the interpretation of earlier studies’ findings, where there was underestimation of disease activity by DAS28-CRP compared to DAS28-ESR, if the lower reporting limit of <10 mg/L was used by the local laboratory.^[19,20]^

RA response to treatment has been improved extensively since the introduction of DMARDs.^[21,22]^ Although we confirm this, it is reasonable to consider the impact of reporting limit of CRP on the calculation of DAS28 and evaluating response to treatment, since reporting lower values of CRP by new methods or reducing the lower limit of CRP will cause lower DAS28 implying better response to treatment. Therefore, the authors conclude that the results of different studies evaluating different treatment modalities should not be compared, if the reporting limits of CRP are unknown.

The limitation of the present study is that the intraindividual biological variation of CRP in patients with RA is not known. This might be lower, higher, or the same as healthy individuals; however, we have used a rather conservative value (low value) of CV-within in our calculations. If CV-within actually is higher in RA, impact on DAS28 will be larger and reporting limits as high as 4 or 5 mg/L might be theoretically acceptable in case of calculation of DAS28. The results of this study have a high degree of generalizability owing to a feasible sample size and broad inclusion criteria and are relevant to all rheumatology departments.

In conclusion, clinicians should be cautious to interpret DAS28 when results of CRP are within normal values, no matter which reporting limit is used by the local laboratory, as intraindividual biological variation of CRP might cause a significant fluctuation in the final DAS28 leading to inaccurate patient classification. It is always advisable to use >1 CRP result and DAS28 calculation before classifying any RA patient.
